# Two Modeling Strategies in Analyzing Clustered Time-to-Event Data: the Strong Heart Family Study

**DOI:** 10.5888/pcd22.240387

**Published:** 2025-03-27

**Authors:** Heather Willmott, Caroline Gochanour, Kai Ding, Jessica Reese, Elisa Lee, Ying Zhang

**Affiliations:** 1 Center for American Indian Health Research, Department of Biostatistics and Epidemiology, Hudson College of Public Health, University of Oklahoma Health Sciences, Oklahoma City; 2 Department of Biostatistics and Epidemiology, Hudson College of Public Health, University of Oklahoma Health Sciences, Oklahoma City

## Abstract

Researchers need applicable tools to analyze and account for familial relatedness when working with family study data. In this brief article, we describe the application of 2 modeling strategies for studying the association between leukocyte telomere length and incident stroke based on data collected in the Strong Heart Family Study: the shared frailty model and the marginal Cox proportional hazards model. Although these modeling strategies are based on different theoretical frameworks, their results were similar. Future simulation study may help us to better understand the limitations and performance of each strategy in a controlled environment.

**Summary**

**What is already known on this topic?**

The shared frailty model has been a popular way of analyzing clustered survival data, though other methods, like the marginal Cox model, handle this data.

**What is added by this report?**

We used data on leukocyte telomere length and stroke to demonstrate that the marginal Cox model produces very similar results to the shared frailty model.

**What are the implications for public health practice?**

The marginal Cox model adds to the toolbox for analyzing clustered survival data in population genetic studies, which investigate the hereditary component of human diseases. Researchers may choose the marginal Cox model when the model will be interpreted at the population level and a robust covariance estimator is required.

## Objective

The Strong Heart Study (SHS) is a cohort study of cardiovascular diseases (CVD) among American Indians living in Arizona, Oklahoma, North Dakota, and South Dakota. In Phase IV of the SHS (also called the Strong Heart Family Study [SHFS]), members of 91 families from 12 tribal communities were recruited and assessed for demographic, clinical, and behavioral characteristics (1,2). Participants have been followed for CVD outcomes to the present day. When analyzing data from the SHFS, we must address relatedness among family members.

The shared frailty model is one approach for analyzing clustered time-to-event data (3). We used it previously to determine the association between leukocyte telomere length (LTL) and cardiometabolic outcomes, such as stroke (4), carotid atherosclerosis (5), and diabetes (6). The marginal Cox proportional hazards model provides another approach to account for familial relatedness in survival data analyses (7). However, its application is less demonstrated in family studies.

In this report, we used both the shared frailty and the marginal Cox proportional hazards models to study the association between LTL and time-to-incident stroke. We hypothesized that results generated by both approaches would be similar. We aimed to illustrate the use of multiple tools for researchers to appropriately analyze family study data.

## Methods

The Cox proportional hazards model (Cox model) is commonly used to identify risk factors that affect survival time among independent participants. To analyze clustered data, the shared frailty model adds a random frailty term to the Cox model, which models the effect of cluster membership on the outcome risk (3). Conversely, the marginal Cox model (7–9) accounts for family relatedness by using a robust sandwich covariance estimator, which makes no distributional assumptions about the model parameters and is consistent even when model assumptions (eg, independence) are violated (10,11). 

Full details about the study design were published previously (1,2). We included data from 4,635 people from the original and family cohorts who were stroke-free at the time of their baseline examinations (1989–1991 and 2001–2003, respectively) and had LTL measurements. Participants were followed through December 31, 2018, for fatal and nonfatal stroke events (12,13), and they all gave informed consent. This study was approved by the institutional review boards of the participating institutions, the participating tribes, and area offices of the Indian Health Service (4).

Summary statistics were generated, and *P* values were obtained by using the χ^2^ test or Mann–Whitney test. Four shared frailty and marginal models were built in the same manner with time to first stroke as the outcome. We first studied the univariable association between age-adjusted LTL (in log quartile) and stroke. We then built 3 multivariable models with demographic (Model 2), behavioral (Model 3), and clinical (Model 4) covariates added to the models sequentially to create our final model. Covariates were chosen based on our previous work and literature review (4). Hazard ratios for each log LTL quartile were obtained. Type III tests assessed the significance of the frailty term. All models were created in SAS, version 9.4 (SAS). 

## Results

Among our 4,635 participants, 2,645 belonged to 87 families, and 1,990 were independent individuals considered as single-member families. Family sizes ranged from 1 to 109 (median, 31). In total, 301 participants experienced incident stroke with a median follow-up time of 16.8 years (interquartile range: 15.0–20.3) (Table 1). Those who had a stroke event were older, had higher blood pressure, and had worse lipid profiles (higher triglyceride, higher total and LDL cholesterol, and lower HDL-cholesterol) than participants free from stroke event during the follow up. The prevalence of atrial fibrillation, diabetes mellitus, and smoking was higher in those with a stroke event than those without a stroke event.

**Table 1:** Baseline Characteristics by Incident Stroke Status^a^

Variables	Total (N = 4,635)	Incident stroke (n = 301)	Stroke-free (n = 4,334)	*P* value^b^
Leukocyte telomere length (LTL)	1.0 (0.9–1.2)	1.0 (0.8–1.4)	1.0 (0.9–1.2)	.85
Age, y	48.2 (36.8–56.5)	56.2 (50.0–63.1)	47.7 (35.7–55.8)	<.001
Sex, male, n (%)	1,900 (41)	120 (40)	1,780 (41)	.68
Phase I Cohort, yes, n (%)	2,369 (51)	237 (79)	2,132 (49)	<.001
Field sites, n (%)				<.001
Arizona	499 (11)	13 (4)	486 (11)	
Oklahoma	1,889 (41)	103 (34)	1,786 (41)	
Dakotas	2,247 (48)	185 (61)	2,062 (48)	
Education, y	12.0 (10.0–14.0)	12.0 (10.0–13.0)	12.0 (10.0–14.0)	<.001
Smoking, yes, n (%)	3,089 (67)	223 (74)	2,866 (66)	.005
Body mass index, kg/m^2^	29.9 (26.2–34.5)	30.0 (26.5–34.3)	29.9 (26.1–34.5)	.77
Atrial fibrillation, yes, n (%)	270 (6)	46 (15)	224 (5)	<.001
Diabetes mellitus, yes, n (%)	1,197 (26)	137 (46)	1,060 (25)	<.001
Systolic blood pressure, mmHg	121.0 (111.0–132.0)	128.0 (117.0–140.0)	121.0 (111.0–132.0)	<.001
Diastolic blood pressure, mmHg	76.0 (69.0–83.0)	77.0 (71.0–84.0)	76.0 (69.0–83.0)	.03
Total cholesterol, mg/dL	186.0 (162.0–211.0)	192.0 (169.0–216.0)	185.0 (162.0–210.0)	<.001
LDL cholesterol, mg/dL	102.0 (83.0–124.0)	106.0 (88.0–130.0)	102.0 (83.0–124.0)	.006
HDL cholesterol, mg/dL	46.0 (39.0–56.0)	44.0 (37.0–54.0)	46.0 (39.0–56.0)	.002
Triglycerides, mg/dL	123 (87.0–179.0)	130.0 (96.0–180.0)	122.0 (86.0–179.0)	.05

Abbreviations: LTL, leukocyte telomere length; LDL, low-density lipoprotein; HDL, high-density lipoprotein.

a Continuous variables are described by using the median (first quartile, third quartile). Categorical variables are described by using as count (percentage). Participants were monitored for stroke events for a median follow-up time of 16.8 years (interquartile range, 15.0–20.3). Values are median (interquartile range) unless otherwise noted.

b Calculated by using the χ^2^ test for categorical variables and the Mann–Whitney test for continuous variables.

Across both the shared frailty and marginal models, point estimates, CIs, and *P* values are almost the same, except for the univariate models that showed about 5%–10% differences (eg, hazards ratio of 0.88 and 0.90 from the frailty model vs 0.83 and 0.98 from the marginal model) (Table 2). For the shared frailty model, the frailty term was significant for all models except Model 1 (*P* = .06), though results for all models were similar to independent Cox models. Both methods showed that after adjustment for demographic, behavioral, and clinical covariates, participants whose LTL was in the third quartile had significantly lower risk of developing a stroke event during the 17-year follow-up period with a hazard ratio of 0.66 (95% CI, 0.46–0.94; *P* value, .02) compared with participants with LTL in the first quartile. Participants with LTL in the second or fourth quartiles did not have significantly different risks of developing a stroke compared with participants with LTL in the first quartile. The shared frailty model and the marginal model generated similar estimates on the same set of data collected in the SHFS.

**Table 2:** The Association Between Log LTL and Time to Incident Stroke, From the Frailty and Marginal Models^a^

Model	Log LTL quartile	Frailty model		Marginal model	
		Hazard ratio (95% CI)	*P* value^b^	Hazard ratio (95% CI)	*P* value^c^
Model 1, univariable model	2 vs 1	0.88 (0.64–1.23)	0.46	0.83 (0.61–1.13)	.24
	3 vs 1	0.53 (0.37–0.77)	<.001	0.54 (0.38–0.75)	<.001
	4 vs 1	0.90 (0.66–1.23)	0.50	0.98 (0.73–1.30)	.87

Model 2, adjusted for demographic covariates^d^	2 vs 1	0.95 (0.69–1.30)	.75	0.95 (0.70–1.30)	.75
	3 vs 1	0.61 (0.43–0.87)	.007	0.61 (0.43–0.86)	.005
	4 vs 1	0.90 (0.67–1.21)	.49	0.90 (0.68–1.19)	.47

Model 3, adjusted for covariates in model 2 plus behavioral covariates^e^	2 vs 1	0.97 (0.71–1.33)	.86	0.97 (0.71–1.33)	.86
	3 vs 1	0.62 (0.44–0.89)	.01	0.62 (0.44–0.88)	.007
	4 vs 1	0.92 (0.69–1.24)	.59	0.92 (0.70–1.22)	.57

Model 4, adjusted for covariates in models 2 and 3 plus clinical covariates^f^	2 vs 1	0.95 (0.69–1.32)	0.77	0.95 (0.69–1.31)	.77
	3 vs 1	0.66 (0.46–0.94)	.02	0.66 (0.46–0.93)	.02
	4 vs 1	0.94 (0.69–1.26)	.67	0.94 (0.70–1.25)	.66

Abbreviations: LTL, leukocyte telomere length.

a For each frailty model, the significance of the frailty term was assessed using type III tests. The frailty term was significant for Model 2 (*P* < .001), Model 3 (*P* < .001), and Model 4 (*P* < .001) but insignificant for Model 1 (*P* .06).

b *P* values calculated by using the Wald test. Significant at *P* < .05.

c *P* values calculated by using the robust Wald test. Significant at *P* < .05.

d Demographic covariates: study site, cohort, and education.

e Behavioral covariates: smoking status and body mass index.

f Clinical covariates: atrial fibrillation, diabetes, systolic and diastolic blood pressure, total, LDL and HDL cholesterol.

## Discussion

Two modeling strategies, the shared frailty model and the marginal Cox proportional hazards model, generated similar estimates in studying the association between LTL and incident stroke based on the same data collected in the SHFS. Although previous studies have used the shared frailty model (4–6), our results show that the less complex marginal Cox model could be considered as a viable alternative for clustered data, such as family or panel data. However, we must consider the advantages and disadvantages of each model when choosing the best model for a situation.

The shared frailty model accounts for the relatedness between family members by introducing a random variable called a frailty to a Cox proportional hazards model (3). Each family is treated as a cluster, and each individual family member is treated as a randomly selected individual from that cluster. One advantage of this model is that the differences between each of the clusters can be easily described (14). In addition, if the frailty term is found to be insignificant, we can reduce our model to an independent Cox model. The shared frailty model yields more efficient estimation when the distribution of the frailty term is modeled correctly. However, this is prone to misspecification because choices for this distribution are limited by software. Coefficients from the shared frailty model should be interpreted as conditional on the unobserved frailty term (7). In contrast, the marginal Cox proportional hazards model uses a robust sandwich covariance estimator to account for the relatedness between family members. A benefit of this model is that the dependence between related observations is unspecified, which allows for greater flexibility in practice because we are not limited by our ability to correctly specify a frailty model (7). However, this model is still somewhat reliant on the specified model and can be affected if the coefficients are heavily biased by unobserved covariates. The marginal model can be interpreted at the population level (7). Both models are useful tools for analyzing survival data from family studies, such as the SHFS. A simulation study of the 2 modeling strategies would be helpful for us to better understand their limitations and performance under a controlled environment. In addition, future studies may consider comparing methods for clustered competing risks data. However, it is beyond the scope of this brief article aiming to demonstrate the application of both methods in analyzing clustered survival data collected from family studies.
